# Experiences of older people in Long-Term Care Institutions in light of the theory of Liquid Modernity

**DOI:** 10.1590/1980-220X-REEUSP-2026-0016en

**Published:** 2026-06-05

**Authors:** Marcos Abelbeck de Oliveira, Márcia Aparecida Padovan Otani, Juliana Ribeiro da Silva Vernasque, Juliane Noack Napoles, Anja Herzog, Maria José Sanches Marin

**Affiliations:** 1 Faculdade de Medicina de Marília, Marília, SP, Brazil.; 2 Brandenburg University of Technology Cottbus-Senftenberg, Cottbus, Brandenburg, Alemanha.

**Keywords:** Homes for the Aged, Aged, Humanization of Assistance, Life Change Events, Institutionalization

## Abstract

**Objective:**

To get to know the experiences of older people in the process of institutionalization.

**Method:**

This is a qualitative study based on interviews with 30 older people residing in three Long-Term Care Facilities for Older People (LTCF) in a municipality in an inland city of the state of São Paulo, Brazil. The analysis was conducted using thematic analysis techniques, in light of the theory of Liquid Modernity, from September to December 2024.

**Results:**

Based on the data analysis, four themes were formulated: Institutionalization due to inability to provide care and as a process imposed by the family; Losses resulting from institutionalization; Suffering and discomfort in institutional living; The institution as a space of care, security, and reconstruction of belonging.

**Conclusion:**

The experiences of institutionalized older people expressed the complexity of care in contemporary times, marked by ambivalent experiences between protection and suffering. The findings indicated a need to improve care practices, invest in staff training, and strengthen public policies that promote humane care, autonomy, and the continued presence of older adults in their social context, whenever possible.

## INTRODUCTION

In the last decades, concern has intensified regarding the accelerated process of population aging observed worldwide. In Brazil, it is estimated that by 2060, older people will represent 25.5% of the population^(1,2)^, configuring a new epidemiological profile. Thus, it should be noted that the transformations resulting from aging tend to increase vulnerability, reduce functional capacity, and favor the emergence of multiple diseases, in addition to intensifying interpersonal conflicts and socioeconomic problems. These factors can compromise living conditions and health, highlighting the need for adequate preparation of services and policies to meet the demands of this group^(1,2)^.

In the Brazilian context, the main support for older people is usually provided by the family, who plays a central role in their care and support for their needs. However, socioeconomic changes, the reduction in family size and structure, the overload of daily tasks, and the scarcity of material or emotional resources often limit the ability to provide adequate care. Given these difficulties, there has been a considerable increase in the demand for Long-Term Care Facilities for older people (LTCFs), which emerges as an alternative to ensure the necessary care and safety for older people^(3)^.

Institutionalization, while an important care alternative, is fraught with challenges involving different dimensions of autonomy and independence. Older people living in LTCFs are isolated from family ties and the relationships in which they built their life history. In addition, they may present some degree of limitation in activities of daily living and, therefore, require continuous care^(4)^.

Rigid routines and standardized care limit older people’s choices, reduce their decision-making capacity, and consequently, their ability to exercise preferences regarding the organization of their daily lives. In this context, they become dependent on the team’s availability to execute decisions, even when they are still capable of making them, revealing social and institutional dependence^(4)^. These consequences are associated with a poorer quality of life, a higher risk of sadness, isolation, and psychological distress^(5)^.

Institutionalized older individuals exhibit a high degree of functional dependence in activities of daily living, particularly those related to personal hygiene^(6)^. Loneliness, which they frequently experience, has negative impacts on their health^(7)^. There is also a correlation between institutionalization and higher levels of depression, anxiety, and low self-esteem, which worsens pre-existing health conditions, makes managing chronic diseases more difficult, and impairs eating habits^(8)^.

Beyond demographic and epidemiological changes, it is essential to understand the institutionalization of older adults within the context of contemporary social transformations. In light of Zygmunt Bauman’s theory of liquid modernity, it is observed that human relationships have become progressively more fragile, unstable, and utilitarian, marked by a logic of disposability^(9)^.

In this scenario, old age, often associated with unproductivity, dependency, and loss of social value, becomes invisible and marginalized. Bauman further describes “moral blindness” as a characteristic phenomenon of liquid modernity, in which the suffering of others ceases to provoke ethical outrage and social mobilization. This perspective helps to understand why situations of neglect, abandonment, and compulsory institutionalization of older people are often normalized, both within the family and in institutional and state spheres. Thus, institutionalization ceases to be merely a response to functional limitations and also begins to reflect the fragility of social bonds in contemporary society^(9,10)^.

Despite the growing number of studies on aging and LTCF, it is observed that much of the scientific production focuses on epidemiological, functional, and organizational aspects of these institutions. Aspects of subjective dimensions remain underexplored, especially from the perspective of the older people themselves, which limits the development of more sensitive and person-centered care practices^(3,4,5,6,7,8)^.

Given this scenario, the aim of this study was to give voice to older people living in LTCF, seeking to broaden knowledge about the subjective dimensions of this process and, thus, contribute to the development of more sensitive, humanized care practices aligned with the needs of these individuals. In this context, the following research question was formulated: What are the experiences of older people in the process of institutionalization in LTCF? Therefore, the objective of the study was to get to know the experiences of older people in the process of institutionalization.

## METHOD

This qualitative study, based on interviews with older people living LTCF, was conducted in accordance with the guidelines of the Consolidated Criteria for Reporting Qualitative Research (COREQ). The results were compiled based on Thematic Analysis (TA), as proposed by Brawn and Clarke^(11)^.

The project was submitted to the Human Research Ethics Committee of the proposing institution and received a favorable opinion no. 6.741.923, CAAE 78071324.4.0000.5413. The older people were informed about the study’s development, in accordance with Resolution 510/2016 of the National Ethics and Research Commission (*CONEP*), and, when in agreement, signed the Informed Consent Form.

The research was conducted in a municipality in an inland city of the state of São Paulo, Brazil. Three LTCF were surveyed. The municipality has a population of 101,409 inhabitants, of which 19,543 are older people, representing approximately 19% of the total population.

The institutions were named A, B and C, which are characterized as civil associations, of a legal nature, philanthropic and non-profit, receiving municipal and state resources, as well as help from the local population. They are registered with the Municipal and State Councils for Older People and have 76, 44, and 47 residents, respectively. The criteria for admission to these institutions are determined by court order, justified by family abandonment, spontaneous demand, or intervention by the Municipal Council for Older People. Some residents are under 60 years of age, which occurs due to dependence for activities of daily living and a lack of social or family support network to ensure survival in the community. Currently, there is a waiting list for the admission of new older people to these institutions.

The inclusion criteria for the research were: being institutionalized for at least six months, being 60 years of age or older, and having preserved communication and cognitive abilities, as assessed using the Mini-Mental State Examination (MMSE). Older individuals diagnosed with dementia and those who were hospitalized or unwell due to health problems at the time of the interview were excluded. The MMSE was applied to all older individuals in a previous phase of the study. Among those who met the established criteria, a draw was held to select the participants. The initial invitation was made by a professional from the LTVF who had a prior relationship with the older person; upon agreement, the researcher was introduced and proceeded to conduct the interview. In cases of refusal, a new draw was held.

The data were collected through semi-structured interviews, by the first author, nurse, master, who indirectly performs professional activities within the institutions, as he works in the municipal health surveillance service at the research site. The interviewer had no prior relationship with the participants and explained the motivations for the research before data collection. In addition, he received prior training on how to conduct the interviews. The script with identification data (sex, age, length of residence in the LTCF and open-ended questions about the reasons for living in a long-term care facility, what has changed in life since moving to a LTCF, the meaning of being institutionalized, and what daily life is like there) was followed.

The interviews were audio recorded, made between September and December 2024, in the three LTVFs, without the presence of other participants or researchers. Furthermore, they had an average duration of 30 minutes and were terminated when data saturation was observed, that is, when data was repeated and the collection of new information did not add anything to the object of study^(12)^. It should be noted that it was not necessary to conduct repeated interviews, that there was no pilot interview, and that the first author took field notes after each interview. The interviews were fully transcribed, immediately after they were conducted, by the interviewer in charge of making them, and the transcripts and results were not returned to the participants. The empirical material was organized manually with the aid of Microsoft Word^®^.

Data analysis was performed using thematic analysis (TA), a qualitative analysis method that aims to understand patterns emerging from the data by identifying themes. The method allows for great flexibility, since the themes are extracted from the data themselves, which for the authors is a creative and reflective process, with the researcher’s subjectivity understood as a necessary resource, as they do not passively emerge from the data. However, it should be emphasized that the approach to coding and developing themes must be rigorous and systematic, considering the researcher’s need for fluency and reflexivity, which must occur with theoretical knowledge and transparency^(11)^.

Furthermore, the development of themes occurs through successive approximations with the data, and thematic analysis is described in six phases, which do not need to be considered linearly, but exhaustive interaction with the data is required to generate rich and complex insights^(13)^.

The first phase of thematic analysis aims to approximate the depth and breadth of the content. Thus, after transcribing the interviews in full, the six authors performed repeated and independent readings of the material, seeking familiarity with the corpus and recording preliminary impressions. The second phase involves producing initial codes from the data, which represent semantic or latent content referring to the most basic segment or element of the data, seeking to identify interesting and significant aspects of the text. In this stage, two researchers proceeded with the initial generation of codes from a line-by-line reading of the interviews, and subsequently, the codes were reviewed, grouped, and refined through online meetings with the other authors. In phase three, called the search for themes, the codes were organized into potential themes, which were discussed collectively and re-evaluated regarding their internal coherence (homogeneity) and distinction from each other (external heterogeneity). In phase four, a time to revisit and refine the themes, a thematic map was developed, seeking to clarify the essence of each theme, aiming to ensure the consistent representation of the dataset. Subsequently, the themes were defined and named, seeking to identify the essence of each one, and the sub-themes were verified, ensuring that they consistently represented the data set. In the final phase, the final analysis was carried out and the report was written. In this report, excerpts from the participants’ statements were selected and incorporated into the analytical narrative, aiming to illustrate and support the interpretation collectively constructed by the team^(11,13)^.

To maintain anonymity, the interviews were coded according to the letter assigned to the institution (A, B, and C), followed by the numerical order in which they were conducted, from 1 to 12.

## RESULTS

Thirty interviews were conducted with institutionalized older people. Of the twelve interviews conducted at institution A, five were with females and seven with males; the length of residence for most interviewees was between 1 and 5 years, and the average age was between 75 and 89 years. At institution B, nine interviews were conducted, with six male and three female participants. Most of the respondents had been residents for between 1 and 5 years and were between 60 and 74 years old. At Institution C, nine interviews were conducted, with four participants being male and five female. Most of the respondents had resided at the institution for more than 10 years and were between 75 and 89 years old, as shown in Chart 1.

**Chart 1:** Sociodemographic profile of institutionalized older people - São Paulo, Brazil, 2025.

Institution	Interview No.	Female	Male	Age range (years)	Residence Time (years)
A	12	5	7	75-89	1-5
B	9	3	6	60-74	1-5
C	9	5	4	75-89	>10

Author’s elaboration, 2025.

Based on the analysis of the data obtained through the interviews, it was possible to formulate four themes: Institutionalization due to inability to provide care and as a process imposed by the family; Losses resulting from institutionalization; Suffering and discomfort in institutional life; The institution as a space of care, safety, and reconstruction of belonging, as presented below.

### Institutionalization Due to Inability to Provide Care and as a Process Imposed by the Family

The older people interviewed indicated that institutionalization occurred when they became dependent on others for activities of daily living and when conditions such as lack of support, family structure, financial constraints, and living alone prevented them from remaining in their own homes. In this context, some were institutionalized aware of their need, while others felt conflicted, deceived, voiceless, and sometimes hurt, as the decision to institutionalize them was made by their families. Furthermore, there were older people who, although they did not want to, accepted the stay, recognizing that their family was unable to meet their needs, as can be seen in the following statements.

*So, they couldn’t make lunch or breakfast on time, so they brought me.* (2B)

*Ah, I was left alone, I lost my wife. You are left alone, and they grabbed me and brought me here. And, I don’t... for myself, I wouldn’t have come.* (A1)

*Yes, my nephew brought me here, I didn’t know either.* (B4)

*You feel bad because they lied to me, you know. Then, they lied to me. That he was taking me to the doctor. They should have told the truth*. (C2)

*That’s why I came here, so you can see., We had to pay the caregiver out of my salary. How was I going to do it?* (5C)

*I lived with my sisters for 8 years, and then they couldn’t handle it anymore* [...] *I’ve reached my limit now, they’re exhausted from doing everything, that’s why I came here* [...]*.* (A9)

### Losses Resulting From Institutionalization

The life of an institutionalized older person is expressed by loss of autonomy and independence; a break with identity and home; a daily routine of mechanized and impoverished practices; and a feeling of confinement and not belonging. The desire to be in their own home and have the autonomy to carry out daily activities according to their own will was a recurring theme in the discourse of older people, which generates discontent and sadness due to living in institutional settings. From this perspective, they pointed to the monotony of daily life at the long-term care facility, describing a routine permeated by inactivity and a lack of meaning. This reveals that the older person perceives a reduction in their prominence and possibilities for choice, as well as a break with their identity and home.

*I think we get stuck. The thing is, I hardly ever go out.* (B8)

*If I were at home, I would eat what I want. Because at home, we do what we want. If I could stay at home, I would.* (A2)

*I don’t consider this place my home, I don’t feel this place as my home* [...] (C8)

*Oh, it’s passing by. You get up...they give you a bath...you have breakfast...you have lunch, you have breakfast...And... you have breakfast again, have lunch...Dinner...see? And then we go to bed.* (A1)

### Suffering and Discomfort in Institutional Settings

Regarding the feelings arising from institutionalization, the accounts revealed emotional suffering marked by loneliness and estrangement from family members; collective environments with stressors and lack of privacy; difficult coexistence with other older people; and low identification with the institutional environment. They claimed that at the LTCF there were very different and ill people, some with mental disorders who were difficult to live with, that the environment was noisy, and that three people in a room was embarrassing and suffocating, leading to isolation, a feeling of inadequacy, and a lack of belonging.

*That’s sad. Being away from family. Because if I tell you it’s a good thing, it’s not true. Sometimes I’m sad, I cry, I have to cry, you know*. (B6)

*Dude, I’m telling you the truth, I’m here, but I’m not happy, you know?* (A4)

*It is an institution for sick older people. So ... Because this is difficult, difficult people. So, I don’t pay attention to my surroundings.* (2C)

*Some of them have mental health issues, it’s difficult. Not that I don’t like them. It doesn’t work*. (B7)

*Then, most of the time I spend inside. I’m not very good at it; it’s a lot of shouting and chaos. Also, it’s all kind of crazy, loud, messy, full of crying and swearing*.(A2)

*That’s because there are three of us in the room. You know what it is. Three in the room. I go crazy without air.* (A5)

### The Institution as a Space for Care, Safety, and Rebuilding of a Sense of Belonging

The older people interviewed valued institutional support, as they achieved improvements in health and well-being from the care provided; the institution provided tranquility and protection in the face of a lack of support and resources from their families, which manifested itself in experiences marked by abandonment, conflict, and suffering; they built new bonds and a sense of belonging to the environment; with this, they got adapted and felt satisfied with the institutional routine. It was observed, therefore, that the institution was considered a path of protection and care, providing security and stability, as it had important resources to maintain survival, such as food at the right time, hygiene care, clean clothes, medicines, distractions like radio and television, in addition to the attention they received from the staff.

*It has improved a lot, because here I have proper food, I sleep peacefully, I have to stay here, it’s better.* (A12)

*Ah...Their treatment, you see? Ah...excellent, you see?* (A1)

*I like that they serve us here at the right time. It’s good. So, food, clean clothes.* (B3)

*Health has also improved considerably. Many good things. Excellent*. (A9)

*I sit here listening to the radio in my wheelchair and I also watch television. I like everything that comes my way. Nothing bothers me.* (C5)

*I have no family. My family are the people here at the shelter.* (B2)

*Because I had no peace, I lived in terrible agony. I couldn’t sleep at night. And my daughter would come home drunk. I kept thinking: what’s going to happen today?* (C4)

*This is my home now. When I lived with my brother and sister-in-law, they would go out and leave me alone at home, thirsty and hungry*. (B6)

*Ah, I used to live with my daughter and we didn’t get along, I found everything difficult, so I thought I’d go live in a nursing home, I came and I liked it. For me, it’s better than at home* [...] (A9)

## DISCUSSION

In the experiences of institutionalized older people, it was observed that entry into the institution occurred under different circumstances, predominantly associated with a process of functional fragility and insufficient family support. Dependence on others for performing activities of daily living, coupled with family burden and scarcity of resources, constitutes a set of factors that frequently culminates in institutionalization^(14)^. Often, this process was not participatory, taking the form of a family decision mediated by concrete limitations in caregiving capacity. Although it may represent a practical solution for the family, institutionalization was experienced by older people as an imposition, accompanied by frustration, resentment, and a feeling of loss of autonomy^(15)^.

In this context, autonomy stood out as a relevant dimension in understanding the experiences reported. Beyond its functional capacity, it is an ethical principle that upholds human dignity. When the decision to institutionalize an older person is made without their participation, it creates a rupture with self-determination that can affect their identity and how they perceive their place in the world. In this respect, the process of institutionalization is not limited to a change of residence, but involves the reconfiguration of social roles, reorganization of daily life, and a redefinition of identity. Therefore, it is necessary for professionals who work with these individuals to have a clear understanding of the meaning of autonomy and how to facilitate it effectively^(16)^.

Institutionalization has a significant impact on the lives of older adults, as it provides residents with clearly defined boundaries and rules that reorganize their daily routines. It initially involves a process of expropriation of roles, loss of self-determination and lack of privacy, followed by the gradual reconstruction of new roles, most of them meticulously regulated by institutional norms. This process can be interpreted as a forced transition between modes of existence, in which the previously constructed identity is partially dismantled to give way to an institutional identity. With the loss of self-determination, some feel frustrated and dissatisfied^(17)^.

The institutional daily routine, as expressed in the interviewees’ statements, frequently involves practices that violate the dignity and rights of older people, which end up being normalized and perceived as necessary for maintaining the institution’s functioning. This naturalization contributes to the older person being seen more as an object of labor than as a human being endowed with uniqueness, which favors the loss of subjectivity^(18)^.

In the context of institutionalization, a strong feeling of not belonging emerged, manifesting as exclusion, rejection, and isolation. These feelings weaken self-esteem, compromising personal fulfillment, the achievement of well-being, and, in some cases, favoring the emergence of psychological suffering and mental illnesses^(19)^. From this perspective, non-belonging expresses a rupture with the social bonds that gave meaning to existence prior to institutionalization.

The findings of this study, which highlighted institutionalization as a frequently imposed process permeated by loss, emotional suffering, and weakening of identity, directly resonated with the assumptions of modernity, as discussed by Zygmunt Bauman. In liquid modernity, social relationships have become more vulnerable, ephemeral, and pragmatic, which directly impacts how society deals with old age and dependency. As social relationships become more fragile and disposable, caring for others, especially those who demand time, patience, and responsibility, tends to be perceived as a burden rather than an ethical commitment^(9,10)^.

In this context, the concept of “the adiaphorization of the human conduct” helps to understand the banalization of the suffering of institutionalized older people. According to Bauman, adiaphorization refers to the displacement of certain actions from the realm of moral responsibility, allowing decisions such as institutionalization without the participation of the older person to be made without generating guilt or deep ethical reflection^(10)^. Thus, decisions such as institutionalization without the participation of the older person are justified as necessary or inevitable, without ethical reflection or in-depth consideration of their subjective consequences. This was expressed in the participants’ statements, who reported being deceived, deprived of choice, and abruptly removed from family life.

Furthermore, the logic of consumerism, productivity, and performance, central to liquid modernity, clashes directly with the needs of aging, which demand continuous care, listening, time, and emotional investment. Those who do not conform to the logic of efficiency end up being socially displaced to spaces of functional segregation. Thus, the older person, no longer corresponding to the ideals of full autonomy and efficiency, comes to be perceived as a surplus body, which contributes to the naturalization of exclusion from the family and social sphere, reinforcing the feelings of non-belonging identified in this study^(9,10)^.

Thus, the process of institutionalization brings many challenges for the older person. The adjustment period becomes almost unbearable, bringing feelings of sadness and abandonment, and can lead to depression^(20)^. In many institutions, the lack of physical infrastructure and human resources, coupled with a purely protectionist approach that offers little encouragement to develop the older person’s potential and freedom of choice, can increase the level of dependency, isolation, and prospects for an active and high-quality life^(21)^.

However, long-term care facilities play an important role in addressing the multiple needs of older people, who do not always have adequate support to maintain their health and lives. This was observed in the statements of the interviewees, who considered that they found better living conditions in the institution compared to previous experiences of scarcity of financial resources or family support. However, this protective function does not eliminate the vulnerabilities that permeate the institutional context. Older institutionalized people have multiple and complex needs, requiring continuous, qualified, and interdisciplinary care. In this scenario, those organizations, especially the ones of a philanthropic nature, face structural, financial, and organizational limitations that directly impact the quality of care provided^(22)^.

The literature indicates that the lack of qualified professionals in LTCF is a central factor in this process, contributing to the deterioration of care and compromising the residents’ quality of life. Moreover, insufficient specialized training and continuing education can contribute to the illness of the workers themselves, who deal with complex and increasing demands, often without adequate support^(23,24)^.

In Brazil, although there are robust public policies, such as the Statute of the Older Person, there is still a lack of concrete actions by the State in providing care to the older population. As a consequence, privately managed LTCF have grown in the country, often operating without proper regulation^(25,26)^.

Unlike what this study and others suggest regarding the shortcomings of LTCFs in Brazil, the findings in Austria serve as an example. In general, the quality of life in homes for older people is high, surpassing, in some aspects, that of older people living in homes with similar social and health characteristics, even considering the need for improvements in care related to autonomy^(27)^.

In this country, older people receive financial assistance according to their degree of dependency, prioritizing home care with the support of informal caregivers. Additionally, older people’s wishes regarding whether or not to be institutionalized are respected^(28)^. The support given to older people and, consequently, the improved living conditions, are also observed in countries such as Finland, Norway, Sweden, and Denmark, given the significant investments made in care services^(29)^.

It is thus clear that improving the quality of life for older people depends on effective investments that result in the implementation of public policies, starting with existing ones, ensuring actions that promote autonomy, social participation, and the valuing of subjectivity and personal identity. Valuing subjectivity acknowledges that human beings are vulnerable. This category includes older people and their families, as well as healthcare professionals who provide care in long-term care facilities^(30)^.

Finally, it is acknowledged that although the present study contributes to reflections on the needs of institutionalized older people, it is limited by the fact that it was carried out in three LTCFs in a single municipality, which limits the generalization of the findings to other locations, since structure, care practices, local policies and residents’ conditions may vary. Furthermore, because these are sensitive issues related to feelings of abandonment, conflict, and suffering, some older people may have chosen not to reveal painful aspects of their experiences.

## CONCLUSION

This study, by giving voice to institutionalized older people, highlighted that institutionalization predominantly occurs in contexts of inability to provide care at home and insufficient family support, and is often experienced as an imposed process marked by losses, especially of autonomy, bonds, and subjectivity. At the same time, it revealed itself as a space of protection and access to basic conditions for survival, configuring an ambivalent experience, situated between suffering and care, loss and security.

The results of this study allowed us to affirm that the institutionalization of older people cannot be understood solely as a consequence of functional dependence or insufficient public policies, but also as an expression of a mode of social organization that weakens bonds, relativizes responsibilities, and trivializes human suffering. This ambivalence reflects the limitations and possibilities of LTCF for the older people in the Brazilian context.

Therefore, the need to strengthen public policies and support networks that expand alternatives to institutional care is reinforced, as well as the improvement of practices in LTCF, with an emphasis on promoting autonomy, active listening, and personcentered care. Investing in the training of professionals and in the construction of humanized institutional environments is an essential step to ensure more dignified lives for older people.

## Data Availability

All the data supporting the results of this study were published in the article itself.
