# Supplementary material for: Experiences of older people in Long-Term Care Institutions in light of the theory of Liquid Modernity
Source: Rev Esc Enferm USP. 2026 Jun 5;60:e20260016. doi: 10.1590/1980-220X-REEUSP-2026-0016en (PMC13246216; doi:10.1590/1980-220X-REEUSP-2026-0016en)
Supplement: Translation

# Translation: Vivências de pessoas idosas em Instituições de Longa Permanência à luz da teoria da Modernidade Líquida

**DOI:** 10.1590/1980-220X-REEUSP-2026-0016pt

**Authors:** Marcos Abelbeck de Oliveira, Márcia Aparecida Padovan Otani, Juliana Ribeiro da Silva Vernasque, Juliane Noack Napoles, Anja Herzog, Maria José Sanches Marin

**Affiliations:** 1 Faculdade de Medicina de Marília, Marília, SP, Brasil.; 2 Brandenburg University of Technology Cottbus-Senftenberg, Cottbus, Brandenburg, Alemanha.

**Keywords:** Instituição de Longa Permanência para Idosos, Idoso, Humanização da Assistência, Acontecimentos que Mudam a Vida, Institucionalização

## Abstract

**Objetivo:**

Conhecer as vivências das pessoas idosas no processo de institucionalização.

**Método:**

Estudo qualitativo, a partir de entrevistas com 30 pessoas idosas residentes em três Instituições de Longa Permanência para Idosos (ILPI) de um município do interior do estado de São Paulo, Brasil. A análise foi realizada com base na técnica de análise temática, à luz da teoria da Modernidade Líquida, de setembro a dezembro de 2024.

**Resultados:**

A partir da análise dos dados foram formuladas quatro temáticas: Institucionalização por incapacidade de cuidado e como processo imposto pela família; Perdas decorrentes da institucionalização; Sofrimento e desconforto na convivência institucional; A instituição como espaço de cuidado, segurança e reconstrução do pertencimento.

**Conclusão:**

As vivências das pessoas idosas institucionalizadas expressaram a complexidade do cuidado na contemporaneidade, marcado por experiências ambivalentes entre proteção e sofrimento. Os achados indicaram a necessidade de qualificar as práticas assistenciais, investir na capacitação das equipes e fortalecer políticas públicas que promovam cuidado humanizado, autonomia e permanência da pessoa idosa no contexto social, sempre que possível.

## INTRODUÇÃO

Nas últimas décadas, intensificou-se a preocupação com o acelerado processo de envelhecimento populacional observado mundialmente. No Brasil, estima-se que, em 2060, as pessoas idosas representarão 25,5% da população^(1,2)^, configurando novo perfil epidemiológico. Assim, destaca-se que as transformações decorrentes do envelhecimento tendem a elevar a vulnerabilidade, reduzir a capacidade funcional e favorecer o surgimento de múltiplas doenças, além de intensificar conflitos interpessoais e problemas socioeconômicos. Esses fatores podem comprometer as condições de vida e saúde, evidenciando a necessidade de preparação adequada dos serviços e políticas para atender às demandas desse grupo^(1,2)^.

No contexto brasileiro, o principal suporte às pessoas idosas costuma ser fornecido pela família, que desempenha papel central no cuidado e apoio às necessidades delas. Entretanto, as transformações socioeconômicas, a redução do tamanho e da estrutura das famílias, a sobrecarga de tarefas cotidianas e a escassez de recursos materiais ou emocionais, muitas vezes, limitam a capacidade de prestar cuidados adequados. Diante dessas dificuldades, tem-se aumentado consideravelmente a procura por Instituição de Longa Permanência para idosos (ILPI), a qual surge como alternativa para garantir atenção e segurança necessária às pessoas idosas^(3)^.

A institucionalização, embora constitua alternativa de cuidado importante, é revestida de desafios que envolvem diferentes dimensões da autonomia e independência. A pessoa idosa que vive em uma ILPI encontra-se afastada de vínculos familiares e das relações nas quais construiu a história de vida dela. Além disso, pode apresentar algum grau de limitação nas atividades da vida diária e, por isso, demanda cuidados contínuos0^(4)^.

A rotina rígida e a padronização dos cuidados limitam as escolhas das pessoas idosas, reduzem a capacidade de tomada de decisão e, consequentemente, de exercer preferências quanto à organização do cotidiano. Neste contexto, passam a depender da disponibilidade da equipe para executar as decisões, quando ainda são capazes de tomá-las, revelando dependência social e institucional^(4)^. Essas consequências estão associadas a pior qualidade de vida, maior risco de tristeza, isolamento e sofrimento psicológico^(5)^.

Pessoas idosas institucionalizadas apresentam elevada dependência funcional em atividades de vida diária, principalmente relacionadas à higiene pessoal^(6)^. A solidão, frequentemente vivenciada por elas, causa impactos negativos à saúde^(7)^. Há, também, relação da institucionalização com maior nível de depressão, ansiedade e baixa autoestima, o que agrava as condições de saúde preexistentes, dificulta o gerenciamento de doenças crônicas e prejudica os hábitos alimentares^(8)^.

Além das transformações demográficas e epidemiológicas, é fundamental compreender a institucionalização da pessoa idosa no contexto das transformações sociais da contemporaneidade. À luz da teoria da modernidade líquida de Zygmunt Bauman, observa-se que as relações humanas se tornaram progressivamente mais frágeis, instáveis e utilitárias, marcadas pela lógica do descarte^(9)^.

Nesse cenário, a velhice, frequentemente associada à improdutividade, à dependência e à perda de valor social, passa a ocupar lugar de invisibilidade e marginalização. Bauman descreve ainda a “cegueira moral” como fenômeno característico da modernidade líquida, na qual o sofrimento do outro deixa de provocar indignação ética e mobilização social. Essa perspectiva contribui para compreender por que situações de negligência, abandono e institucionalização compulsória das pessoas idosas são frequentemente naturalizadas, tanto no âmbito familiar quanto nas esferas institucionais e estatais. Assim, a institucionalização deixa de ser apenas uma resposta às limitações funcionais e passa a expressar, também, reflexo das fragilidades dos vínculos sociais na sociedade contemporânea^(9,10)^.

Apesar do crescente número de estudos sobre o envelhecimento e sobre ILPI, observa-se que grande parte da produção científica se concentra em aspectos epidemiológicos, funcionais e organizacionais dessas instituições, sendo que aspectos das dimensões subjetivas permanecem ainda pouco explorados, especialmente quando se trata da perspectiva da própria pessoa idosa, o que limita a construção de práticas assistenciais mais sensíveis e centradas na pessoa^(3,4,5,6,7,8)^.

Diante desse panorama, buscou-se dar voz às pessoas idosas que vivem em ILPI, visando ampliar o conhecimento sobre as dimensões subjetivas desse processo e, assim, contribuir para construção de práticas de cuidado mais sensíveis, humanizadas e alinhadas às necessidades dessas pessoas. Neste contexto, formulou-se a seguinte pergunta de pesquisa: Quais são as vivências das pessoas idosas no processo de institucionalização em ILPI? Logo, objetivou-se conhecer as vivências das pessoas idosas no processo de institucionalização.

## MÉTODO

Estudo de abordagem qualitativa, a partir de entrevistas com pessoas idosas residentes em ILPI, conduzido conforme as diretrizes do *Consolidated Criteria for Reporting Qualitative Research* (COREQ). Os resultados foram compilados com base na Análise Temática (AT), proposta por Brawn e Clarke^(11)^.

O projeto foi submetido ao Comitê de Ética e Pesquisa com Seres Humanos da instituição proponente e obteve parecer favorável nº 6.741.923, CAAE 78071324.4.0000.5413. As pessoas idosas foram informadas sobre o desenvolvimento do estudo, em conformidade com a Resolução 510/2016 da Comissão Nacional de Ética e Pesquisa (CONEP) e, quando de acordo, assinaram o Termo de Consentimento Livre e Esclarecido.

A pesquisa foi realizada em município do interior do estado de São Paulo, Brasil. Foram pesquisadas três ILPI. O município conta com população de 101.409 habitantes, sendo que 19.543 são pessoas idosas, ou seja, aproximadamente 19% da população geral.

As Instituições foram denominadas A, B e C, as quais se caracterizam como associação civil, de natureza jurídica, filantrópica e sem fins lucrativos, recebem recursos municipais e estaduais, assim como ajuda da população local. Mantêm registro no Conselho de Idosos Municipal e Estadual e contam com 76, 44 e 47 residentes, respectivamente. Os critérios para admissão nessas instituições ocorrem por determinação judicial, justificada por abandono familiar, demanda espontânea ou por intervenção do Conselho Municipal do Idoso, sendo que alguns dos residentes têm idade inferior a 60 anos, o que ocorre por dependência para as atividades de vida diária e falta de rede de apoio social ou familiar para garantir a sobrevivência na comunidade. Atualmente, há fila de espera para o acolhimento de novas pessoas idosas nessas instituições.

Os critérios de inclusão na pesquisa foram: estar institucionalizado há, no mínimo, seis meses, ter 60 anos ou mais, e capacidade de comunicação e cognitiva preservadas, avaliadas a partir do Miniexame do Estado Mental (MEEM). Foram excluídas as pessoas idosas com diagnóstico de demência e aquelas que, no momento da entrevista, encontravam-se hospitalizadas ou indispostas por algum problema de saúde. O MEEM foi aplicado à totalidade das pessoas idosas em etapa anterior do estudo. Entre aquelas que atenderam aos critérios estabelecidos, realizou-se sorteio para seleção dos participantes. O convite inicial foi feito por um profissional da ILPI com vínculo prévio com a pessoa idosa; mediante concordância, o pesquisador era apresentado e procedia à realização da entrevista. Nos casos de recusa, procedeu-se a novo sorteio.

Os dados foram coletados por meio de entrevistas semiestruturadas, pelo primeiro autor, enfermeiro, mestre, que exerce atividades profissionais indiretamente nas instituições, pois atua no serviço municipal de vigilância sanitária do local da pesquisa. O entrevistador não tinha relacionamento com os participantes e explicou as motivações para a pesquisa antes da coleta de dados. Além disso, recebeu treinamento prévio para condução das entrevistas. Seguiu-se roteiro com dados de identificação (sexo, idade, tempo de residência na ILPI e questões abertas sobre os motivos de estar residindo na ILPI, o que mudou na vida após ir para a ILPI, o significado de estar institucionalizado, como é o cotidiano nesse local).

As entrevistas foram áudio gravadas, realizadas no período de setembro a dezembro de 2024, nas três ILPI, sem a presença de outros participantes ou pesquisadores. Ademais, tiveram duração média de 30 minutos e foram encerradas quando se observou saturação dos dados, ou seja, no momento em que houve repetição dos dados e a coleta de novas informações não trouxe acréscimos ao objeto de estudo^(12)^. Ressalta-se que não foi necessário realizar entrevistas repetidas, que não houve entrevista piloto, e que o primeiro autor realizou notas de campo após cada entrevista. As entrevistas foram transcritas na íntegra, imediatamente após sua realização, pelo próprio entrevistador responsável por sua condução e não houve devolução das transcrições e resultados aos participantes. O material empírico foi organizado manualmente com o auxílio do *Microsoft Word^®^.*

A análise dos dados foi realizada por meio da análise temática AT que consiste em método de análise qualitativo que se propõe a compreender os padrões oriundos dos dados por meio da identificação de temas. O método permite grande flexibilidade, uma vez que os temas são extraídos dos próprios dados, o que para os autores se trata de um processo criativo e reflexivo, sendo a subjetividade do pesquisador entendida como recurso necessário, pois eles não emergem passivamente dos dados. Destaca-se, entretanto, que a abordagem de codificação e desenvolvimento dos temas deve ser rigorosa e sistemática, considerando a necessidade de fluidez e reflexibilidade do pesquisador, o que precisa ocorrer com conhecimento teórico e transparência^(11)^.

Acrescenta-se que a elaboração dos temas ocorre por meio de sucessivas aproximações com os dados e descreve-se a análise temática em seis fases, as quais não precisam ser consideradas linearmente, mas é necessária a exaustividade na interação com os dados, para que sejam geradas percepções ricas e complexas^(13)^.

A primeira fase da análise temática visa aproximação da profundidade e amplitude do conteúdo. Assim, após a transcrição na íntegra das entrevistas, os seis autores realizaram leituras repetidas e independentes do material, buscando familiarização com o corpus e registrando impressões preliminares. A segunda fase envolve a produção de códigos iniciais a partir dos dados, sendo que estes representam um conteúdo semântico ou latente que se refere ao segmento ou elemento mais básico do dado, buscando identificar aspectos interessantes e significativos do texto. Nesta etapa, dois pesquisadores procederam à geração inicial dos códigos a partir da leitura linha a linha das entrevistas e, na sequência, os códigos foram revistos, agrupados e refinados, por meio de reuniões online com os demais autores. Na fase três, denominada procura por temas, os códigos foram organizados em temas potenciais, os quais foram discutidos coletivamente e reavaliados quanto à sua coerência interna (homogeneidade) e distinção entre si (heterogeneidade externa). Na fase quatro, momento de revisitar e refinar os temas, elaborou-se um mapa temático, buscou-se explicitar a essência de cada tema, visando assegurar a representatividade de forma consistente do conjunto de dados. Na sequência, os temas foram definidos e nomeados, na busca de identificar a essência de cada um e verificados os subtemas, assegurando que representassem consistentemente o conjunto dos dados. Na última fase, realizou-se a análise final e a escrita do relatório. Neste relatório, os excertos das falas dos participantes foram selecionados e incorporados à narrativa analítica, visando ilustrar e sustentar a interpretação constituída coletivamente pela equipe^(11,13)^.

Para manter o anonimato, as entrevistas foram codificadas de acordo com a letra designada para a instituição (A, B e C), seguida da ordem numérica de realização de 1 a 12.

## RESULTADOS

Foram realizadas trinta entrevistas com pessoas idosas institucionalizadas. Das doze entrevistas realizadas na instituição A, cinco foram do sexo feminino e sete do sexo masculino, o tempo de residência da maioria dos entrevistados era entre 1 e 5 anos e a média de idade entre 75 e 89 anos. Na instituição B, foram realizadas nove entrevistas, sendo seis participantes do sexo masculino e três do sexo feminino. A maioria dos entrevistados era residente entre 1 e 5 anos e tinha a média de idade entre 60 e 74 anos. Na Instituição C, foram realizadas nove entrevistas, sendo quatro pessoas do sexo masculino e cinco do sexo feminino. A maioria dos entrevistados residia na instituição há mais de 10 anos e tinha a média de idade entre 75 e 89 anos, conforme Quadro 1.

**Quadro 1:** Perfil sociodemográfico das pessoas idosas institucionalizadas – São Paulo, Brasil, 2025.

Instituição	Nº Entrev.	Sexo Fem.	Sexo Masc.	Faixa Etária (anos)	Tempo Residência (anos)
A	12	5	7	75–89	1–5
B	9	3	6	60–74	1–5
C	9	5	4	75–89	>10

Elaboração própria, 2025.

A partir da análise dos dados obtidos por meio das entrevistas, foi possível a formulação de quatro temáticas: Institucionalização por incapacidade de cuidado e como processo imposto pela família; Perdas decorrentes da institucionalização; Sofrimento e desconforto na convivência institucional; A instituição como espaço de cuidado, segurança e reconstrução do pertencimento, conforme se apresentam a seguir.

### Institucionalização por Incapacidade de Cuidado e como Processo Imposto pela Família

As pessoas idosas entrevistadas apontaram que a institucionalização ocorreu a partir do momento em que se tornaram dependentes para as atividades de vida diária e em que condições, como a falta de apoio, estrutura familiar, condições financeiras, além do fato de morar sozinho, impediram-nos de continuar em seus lares. Neste contexto, alguns foram institucionalizados conscientes da necessidade, enquanto outros se sentiam contrariados, enganados, com sentimento de falta de voz e, por vezes, magoados, pois a decisão de institucionalização ocorreu pelos familiares. Ademais, existiam pessoas idosas que, embora não desejassem, aceitaram a permanência, por reconhecer que a família não conseguia atender às necessidades deles, conforme se observa nas falas a seguir.

*Então, eles não podiam fazer o almoço, café na hora, aí trouxeram eu.* (2B)

*Ah, eu fiquei sozinho, perdi a esposa. Você fica sozinho e elas me pegaram e trouxeram para cá. E, eu não... por mim, eu não tinha vindo.* (A1)

*É, o meu sobrinho que trouxe eu, eu não sabia também.* (B4)

*Você fica sentido por causa que eles mentiram para mim, sabe. Aí, mentiram para mim. Que vinha trazer eu no médico. Tinha que ter falado a verdade.* (C2)

*Daí, eu vim aqui porque, para o senhor ver, nós tínhamos que pagar a cuidadora do meu salário. Como é que ia fazer?* (5C)

*Eu morei 8 anos com as minhas irmãs, daí, elas não estavam mais dando conta [...] eu tenho meu limite agora, elas esgotaram de fazer tudo, por isso que eu vim para cá [...].* (A9)

### Perdas Decorrentes da Institucionalização

A vida da pessoa idosa institucionalizada expressa-se por perda da autonomia e independência; ruptura com a identidade e com o lar; cotidiano com rotinas mecanizadas e empobrecidas; sentimento de confinamento e não pertencimento. O desejo de estar na própria casa e ter autonomia para realizar as atividades do cotidiano, de acordo com a própria vontade foi uma manifestação presente no discurso das pessoas idosas, o que gera descontentamento e tristeza por viverem institucionalizados. Nesta perspectiva, apontaram para monotonia do cotidiano da ILPI, descrevendo rotina permeada pela inatividade e falta de significado. Revela-se, assim, que a pessoa idosa percebe a redução do protagonismo e das possiblidades de escolhas, bem como a ruptura com a identidade e o lar.

*Acho que fica preso. É que eu não saio para lugar nenhum quase.* (B8)

*Se eu estivesse na minha casa, aí eu comeria aquilo que eu quero. Porque em casa, a gente faz o que a gente quer. Se eu pudesse ficar na minha casa, eu ficava.* (A2)

*Eu não considero aqui minha morada, eu não sinto aqui como minha morada [...]* (C8)

*Ah, está passando. Você levanta...eles dão banho...você toma café...você almoça, você toma café...E....toma café de novo, almoça...A janta...viu? E depois vai dormir.* (A1)

### Sofrimento e Desconforto na Convivência Institucional

Quanto aos sentimentos decorrentes da institucionalização, as falas mostraram o sofrimento emocional marcado pela solidão e pelo distanciamento dos familiares; ambientes coletivos com fatores estressores e falta de privacidade; convivência difícil com outras pessoas idosas; e baixa identificação com o ambiente institucional. Alegaram que na ILPI existiam pessoas muito diferentes e doentes, com transtornos mentais, de difícil convivência, o ambiente era barulhento e que o fato de ficar em três no quarto era constrangedor e sufocante, o que levava ao distanciamento, percepção de inadequação e falta de pertencimento ao local.

*É triste. Ficar longe da família. Porque se eu falar para o senhor que é boa coisa, não é. Tem vez que ando triste, choro, eu tenho que chorar, sabe.* (B6)

*Cara, eu falo a verdade, eu estou aqui, mas não estou contente, sabe?* (A4)

*É uma instituição de idosos doentes. Então... Devido a isso ser difícil, pessoas difíceis. Então, eu não presto atenção no ambiente.* (2C)

*Tem alguns que têm problema mental, é difícil. Não que eu não goste deles. Não dá certo.* (B7)

*Aí, a maior parte do tempo eu fico lá dentro. Não me dou muito com jeito, é um griteiro, uma bagunça. Também, é tudo meio maluco, griteiro, bagunça, choradeira, uma xingação.* (A2)

*É que eu estou em três no quarto. Você sabe o que é. Três no quarto. Eu fico maluco sem ar.* (A5)

### A Instituição Como Espaço de Cuidado, Segurança e Reconstrução do Pertencimento

As pessoas idosas entrevistadas valorizavam o apoio institucional, uma vez conseguiam melhorias na saúde e no bem-estar a partir dos cuidados ofertados; a instituição proporcionava tranquilidade e proteção frente à falta de suporte e de condições das famílias, o que se revelou em vivências marcadas por abandono, conflitos e sofrimento; eles construíram novos vínculos e sentimento de pertencimento ao ambiente; com isso, adaptavam-se e sentiam satisfação com a rotina institucional. Observou-se, assim, que a instituição era considerada como um caminho de proteção e cuidado, oferecia segurança, estabilidade, pois contava com importantes recursos para manter a sobrevivência, como alimentação na hora certa, cuidados de higiene, roupa lavada, medicamentos, distração como rádio e televisão, além da atenção que recebiam dos funcionários.

*Melhorou bastante, pois aqui tenho alimentação correta, durmo tranquilo, tem que ficar aqui que é melhor.* (A12)

*Ah...O tratamento deles, viu? Ah...excelente, viu? (A1)*

*Gosto de eles servir a gente aqui na hora certa. É bom. Então, comida, roupa lavada.* (B3)

*Para a saúde, também melhorou bastante. Muitas coisas boas. Excelente.* (A9)

*Eu fico aqui ouvindo rádio na cadeira de rodas e também vejo televisão. Tudo que vem eu gosto. Nada incomoda eu.* (C5)

*Eu não tenho família. A minha família são as pessoas aqui do abrigo.* (B2)

*Porque eu não tinha paz, eu vivia numa agonia terrível. Eu não conseguia dormir à noite. E minha filha chegava bêbada. Ficava pensando: o que vai acontecer hoje?* (C4)

*Aqui é minha casa agora, quando morava com meu irmão e cunhada, eles saiam e deixavam sozinha dentro de casa com sede e com fome.* (B6)

*Ah, eu morava com minha filha e não me dava bem, achava tudo difícil para mim, por isso, pensei vou morar no Asilo, vim e gostei. Para mim, é melhor do que em casa [...]* (A9)

## DISCUSSÃO

Nas vivências das pessoas idosas institucionalizadas, observou-se que a entrada na instituição ocorria em diferentes circunstâncias, predominantemente associadas a processo de fragilização funcional e insuficiência de suporte familiar. A dependência para realizar as atividades de vida diária, somada à sobrecarga familiar e à escassez de recursos, constitui conjunto de fatores que frequentemente culmina na institucionalização^(14)^. Muitas vezes, esse processo não foi participativo, configurando-se como uma decisão familiar mediada por limites concretos na capacidade de cuidado. Embora possa representar uma solução prática para a família, a institucionalização foi vivida pelas pessoas idosas como imposição, acompanhada de frustração, mágoa e sensação de perda da autonomia^(15)^.

Nesse contexto, a autonomia destacou-se como dimensão relevante na compreensão das vivências relatadas. Para além da capacidade funcional, trata-se de um princípio ético que sustenta a dignidade humana. Quando a decisão pela institucionalização ocorre sem a participação da pessoa idosa, instaura-se ruptura com a autodeterminação que pode repercutir na identidade e na forma como o sujeito percebe o lugar no mundo. Nesse sentido, o processo de institucionalização não se restringe à mudança de residência, mas envolve a reconfiguração de papéis sociais, reorganização do cotidiano e redimensionamento da identidade. Assim, faz-se necessário que os profissionais que atendem a essas pessoas tenham claro o significado de autonomia e como facilitá-la de forma eficaz^(16)^.

A institucionalização representa grande impacto na vida da pessoa idosa, visto que proporciona aos residentes limites claramente definidos e regras que reorganizam o cotidiano. Envolve, inicialmente, processo de desapropriação de papéis, perda de autodeterminação e falta de privacidade e, na sequência, reconstrução gradual de novos papéis, a maioria deles minuciosamente regulados por normas da instituição. Tal processo pode ser interpretado como uma transição forçada entre modos de existência, na qual a identidade anteriormente construída é parcialmente desarticulada para dar lugar a uma identidade institucional. Com a perda da autodeterminação, alguns se sentem frustrados e descontentes^(17)^.

O cotidiano institucional, conforme expresso nas falas dos entrevistados, frequentemente envolve práticas que violam a dignidade e os diretos das pessoas idosas, as quais acabam sendo naturalizadas e percebidas como necessárias para manutenção do funcionamento da instituição. Essa naturalização contribui para que a pessoa idosa seja vista mais como um objeto de trabalho do que como um ser humano dotado de singularidade, o que favorece a perda da subjetividade^(18)^.

No contexto da institucionalização, destacou-se forte sentimento de não pertencimento, que se manifesta como exclusão, rejeição e isolamento. Esses sentimentos fragilizam a autoestima, comprometendo a realização pessoal, o alcance do bem-estar e, em alguns casos, favorecendo o surgimento de sofrimento psicológico e doenças mentais^(19)^. O não pertencimento, nesta perspectiva, expressa uma ruptura com os vínculos sociais que conferiram sentido à existência anterior à institucionalização.

Os achados deste estudo, que evidenciaram a institucionalização como processo frequentemente imposto, permeado por perdas, sofrimento emocional e fragilização da identidade, dialogaram diretamente com os pressupostos da modernidade, conforme discutida por Zygmunt Bauman. Na modernidade líquida, as relações sociais tornaram-se mais vulneráveis, efêmeras e pragmáticas, o que impacta diretamente a forma como a sociedade lida com a velhice e dependência. À medida em que as relações sociais se tornam mais frágeis e descartáveis, o cuidado com o outro, especialmente com aquele que demanda tempo, paciência e responsabilidade, tende a ser percebido como um fardo, e não como um compromisso ético^(9,10)^.

Nesse contexto, o conceito de “adiaforização da conduta humana” contribui para compreender a banalização do sofrimento das pessoas idosas institucionalizadas. A adiaforização, segundo Bauman, refere-se ao deslocamento de determinadas ações do campo da responsabilidade moral, permitindo que decisões como a institucionalização sem participação da pessoa idosa sejam tomadas sem que gerem culpa ou reflexão ética profunda^(10)^. Assim, decisões como a institucionalização sem participação da pessoa idosa passam a ser justificadas como necessárias ou inevitáveis, sem que haja reflexão ética e aprofundamento sobre suas consequências subjetivas. Isso se expressou nas falas dos participantes que relataram ter sido enganados, privados de escolha e afastados abruptamente do convívio familiar.

Além disso, a lógica do consumismo, da produtividade e da performance, centrais na modernidade líquida, entra em choque direto com as necessidades do envelhecimento, que demandam cuidado contínuo, escuta, tempo e investimento emocional. Aquele que não corresponde à lógica da eficiência passa a ser socialmente deslocado para espaços de segregação funcional. Assim, a pessoa idosa, ao não corresponder mais aos ideais de autonomia plena e eficiência, passa a ser percebida como um corpo excedente, o que contribui para naturalização da exclusão do espaço familiar e social, reforçando os sentimentos de não pertencimento identificados neste estudo^(9,10)^.

Assim, o processo de institucionalização traz para a pessoa idosa muitos desafios. O período de adaptação torna-se quase que insuportável, trazendo sentimentos de tristeza e abandono, podendo levá-la à depressão^(20)^. Em muitas instituições, a carência de estrutura física e recursos humanos, aliada a uma assistência puramente protecionista, com pouco incentivo às potencialidades da pessoa idosa e à liberdade de escolha dela, pode aumentar o grau de dependência, isolamento e de perspectiva para uma vida ativa e com qualidade^(21)^.

Entretanto, as ILPI desempenham importante papel diante das múltiplas necessidades apresentas pelas pessoas idosas, que nem sempre contam com suporte adequado para manutenção da saúde e da vida, assim como foi observado na fala dos entrevistados que consideram que na instituição encontraram melhores condições de sobrevivência, quando comparado com as vivências anteriores de escassez de recursos financeiros ou de apoio familiar. Contudo, essa função protetiva não elimina as vulnerabilidades que atravessam o contexto institucional. As pessoas idosas residentes apresentam demandas múltiplas e complexas, exigindo cuidado contínuo, qualificado e interdisciplinar. Neste cenário, principalmente aquelas de caráter filantrópico, enfrentam limitações estruturais, financeiras e organizacionais que repercutem diretamente na qualidade do cuidado ofertado^(22)^.

A literatura aponta que a carência de qualificação dos profissionais nas ILPI constitui fator central nesse processo, contribuindo para precarização do cuidado e comprometimento da qualidade de vida dos residentes. Além disso, a insuficiência de formação específica e de educação permanente pode favorecer o adoecimento dos próprios trabalhadores que lidam com demandas complexas e crescentes, muitas vezes, sem o suporte adequado^(23,24)^.

No Brasil, embora existam políticas públicas robustas, como o Estatuto da Pessoa Idosa, ainda se observa a insuficiência de ações concretas do Estado na oferta de cuidados à população idosa. Como consequência, as Instituições de Longa Permanência para Idosos (ILPI) de gestão privada têm crescido no país, muitas vezes, operando sem a devida regulamentação^(25,26)^.

Diferentemente do que aponta o presente estudo e outros sobre as deficiências das ILPI no Brasil, cita-se, como exemplo, o que foi constado na Áustria, pois, no geral, a qualidade de vida em lares de pessoas idosas é elevada, superando, em alguns aspectos, pessoas idosas que vivem no domicílio com características sociais e de saúde semelhantes, mesmo considerando a necessidade de melhoria nos cuidados relacionados à autonomia^(27)^.

Nesse país, as pessoas idosas recebem auxílio financeiro de acordo com o grau de dependência, priorizando o cuidado domiciliar com apoio de cuidadores informais. Ademais, é respeitado o desejo da pessoa idosa em ser institucionalizado ou não^(28)^. O suporte dado à pessoa idosa e, consequentemente, as melhores condições de vida, também são observados em países como Finlândia, Noruega, Suécia e Dinamarca, visto que foram realizados investimentos elevados em serviços de cuidado^(29)^.

Resgata-se, assim, que a melhoria da qualidade de vida das pessoas idosas depende de investimentos efetivos que resultem na implementação de políticas públicas, iniciando pelas existentes, assegurando ações que promovam autonomia, participação social e valorização da subjetividade e identidade pessoal. A valorização da subjetividade reconhece que os seres humanos são vulneráveis. Esta categoria inclui pessoas idosas e familiares, bem como os profissionais de saúde que prestam cuidados em ILPI^(30)^.

Por fim, reconhece-se que embora o presente estudo traga contribuições para reflexões acerca das necessidades de pessoas idosas institucionalizadas, apresenta-se como limitação o fato de ter sido realizado em três ILPI de um único município, o que limita a generalização dos achados para outras localidades, já que estrutura, práticas de cuidado, políticas locais e condições dos residentes podem variar. Além disso, por se tratar de abordagem de questões sensíveis que se relacionam com a sensação de abandono, conflitos e sofrimento, algumas pessoas idosas podem ter optado por não revelar aspectos dolorosos das vivências delas.

## CONCLUSÃO

O presente estudo, ao dar voz às pessoas idosas institucionalizadas, evidenciou que a institucionalização ocorre, predominantemente, em contextos de incapacidade para o cuidado no domicílio e insuficiência de suporte familiar, sendo frequentemente vivida como processo imposto e marcado por perdas, especialmente de autonomia, vínculos e subjetividade. Ao mesmo tempo, revelou-se como espaço de proteção e acesso a condições básicas de sobrevivência, configurando experiência ambivalente, situada entre sofrimento e cuidado, perda e segurança.

Os resultados deste estudo permitiram afirmar que a institucionalização da pessoa idosa não pode ser compreendida apenas como consequência da dependência funcional ou da insuficiência de políticas públicas, mas também como expressão de um modo de organização social que fragiliza vínculos, relativiza responsabilidades e banaliza o sofrimento humano. Tal ambivalência reflete os limites e as possibilidades das ILPI no contexto brasileiro.

Diante disso, reforça-se a necessidade de fortalecimento das políticas públicas e das redes de apoio que ampliem alternativas ao cuidado institucional, bem como a qualificação das práticas nas ILPI, com ênfase na promoção da autonomia, na escuta ativa e no cuidado centrado na pessoa. Investir na formação dos profissionais e na construção de ambientes institucionais humanizados constitui passo essencial para assegurar vivências mais dignas às pessoas idosas.

## Data Availability

Todo o conjunto de dados que dá suporte aos resultados deste estudo foi publicado no próprio artigo.

# Translation: Experiencias de ancianos en instituciones de cuidados a largo plazo a la luz de la teoría de la modernidad líquida

**DOI:** 10.1590/1980-220X-REEUSP-2026-0016es

**Authors:** Marcos Abelbeck de Oliveira, Márcia Aparecida Padovan Otani, Juliana Ribeiro da Silva Vernasque, Juliane Noack Napoles, Anja Herzog, Maria José Sanches Marin

**Affiliations:** 1 Faculdade de Medicina de Marília, Marília, SP, Brasil.; 2 Brandenburg University of Technology Cottbus-Senftenberg, Cottbus, Brandenburg, Alemanha.

**Keywords:** Hogares para Ancianos, Anciano, Humanización de la Atención, Acontecimientos que Cambian la Vida, Institucionalización

## Abstract

**Objetivo:**

Conocer las experiencias de los ancianos en el proceso de institucionalización.

**Método:**

Este es un estudio cualitativo basado en entrevistas con 30 ancianos que residen en tres residencias de larga estancia para ancianos en un municipio del interior del estado de São Paulo, Brasil. El análisis se realizó utilizando técnicas de análisis temático, a la luz de la teoría de la modernidad líquida, desde septiembre hasta diciembre de 2024.

**Resultados:**

A partir del análisis de datos, se formularon cuatro temas: Institucionalización debido a la incapacidad de brindar cuidados y como un proceso impuesto por la familia; Pérdidas resultantes de la institucionalización; Sufrimiento e incomodidad en la vida institucional; La institución como espacio de cuidado, seguridad y reconstrucción de la pertenencia.

**Conclusión:**

Las experiencias de los ancianos institucionalizados pusieron de manifiesto la complejidad de los cuidados en la actualidad, marcado por vivencias ambivalentes entre la protección y el sufrimiento. Los halazgos indicaron la necesidad de mejorar las prácticas de atención, invertir en la capacitación del personal y fortalecer las políticas públicas que promuevan una atención humana, la autonomía y la presencia continua de los adultos mayores en su contexto social, siempre que sea posible.

## INTRODUCCIÓN

En las últimas décadas, se ha intensificado la preocupación por el acelerado proceso de envejecimiento de la población observado a nivel mundial. En Brasil, se estima que, en 2060, las personas mayores representarán el 25,5% de la población^(1,2)^, configurando un nuevo perfil epidemiológico. Así, cabe destacar que las transformaciones derivadas del envejecimiento tienden a aumentar la vulnerabilidad, reducir la capacidad funcional y favorecer la aparición de múltiples enfermedades, además de intensificar los conflictos interpersonales y los problemas socioeconómicos. Estos factores pueden comprometer las condiciones de vida y la salud, lo que pone de manifiesto la necesidad de una preparación adecuada de los servicios y las políticas para atender las demandas de este grupo^(1,2)^.

En el contexto brasileño, el principal apoyo a las personas mayores suele ser proporcionado por la familia, que desempeña un papel central en el cuidado y la atención de sus necesidades. Sin embargo, las transformaciones socioeconómicas, la reducción del tamaño y la estructura de las familias, la sobrecarga de tareas cotidianas y la escasez de recursos materiales o emocionales, a menudo limitan la capacidad de brindar cuidados adecuados. Ante estas dificultades, ha aumentado considerablemente la demanda de Instituciones de Larga Permanencia para personas mayores (ILPI en portugués), que surgen como alternativa para garantizar la atención y la seguridad necesarias a las personas mayores^(3)^.

La institucionalización, aunque constituye una alternativa de cuidado importante, está plagada de desafíos que involucran diferentes dimensiones de la autonomía y la independencia. La persona mayor que vive en una ILPI se encuentra alejada de los vínculos familiares y de las relaciones en las que construyó su historia de vida. Además, puede presentar algún grado de limitación en las actividades de la vida diaria y, por ello, requiere cuidados continuos^(4)^.

La rutina rígida y la estandarización de los cuidados limitan las opciones de las personas mayores, reducen su capacidad de tomar decisiones y, en consecuencia, de ejercer sus preferencias en cuanto a la organización de la vida cotidiana. En este contexto, pasan a depender de la disponibilidad del equipo para ejecutar las decisiones, cuando aún son capaces de tomarlas, lo que revela una dependencia social e institucional^(4)^. Estas consecuencias se asocian con una peor calidad de vida, mayor riesgo de tristeza, aislamiento y sufrimiento psicológico^(5)^.

Las personas mayores institucionalizadas presentan una elevada dependencia funcional en las actividades de la vida diaria, principalmente en lo relacionado con la higiene personal^(6)^. La soledad, que suelen experimentar, causa impactos negativos en la salud^(7)^. Existe, además, una relación entre la institucionalización y un mayor nivel de depresión, ansiedad y baja autoestima, lo que agrava las condiciones de salud preexistentes, dificulta el manejo de enfermedades crónicas y perjudica los hábitos alimentarios^(8)^.

Además de los cambios demográficos y epidemiológicos, es fundamental comprender la institucionalización de las personas mayores en el contexto de las transformaciones sociales de la contemporaneidad. A la luz de la teoría de la modernidad líquida de Zygmunt Bauman, se observa que las relaciones humanas se han vuelto progresivamente más frágiles, inestables y utilitarias, marcadas por la lógica del descarte^(9)^.

En este escenario, la vejez, frecuentemente asociada a la improductividad, la dependencia y la pérdida de valor social, pasa a ocupar un lugar de invisibilidad y marginación. Bauman describe además la “ceguera moral” como un fenómeno característico de la modernidad líquida, en la que el sufrimiento del otro deja de provocar indignación ética y movilización social. Esta perspectiva ayuda a comprender por qué las situaciones de negligencia, abandono e institucionalización compulsoria de las personas mayores suelen naturalizarse, tanto en el ámbito familiar como en las esferas institucionales y estatales. Así, la institucionalización deja de ser solo una respuesta a las limitaciones funcionales y pasa a expresar, también, un reflejo de las fragilidades de los vínculos sociales en la sociedad contemporánea^(9,10)^.

A pesar del creciente número de estudios sobre el envejecimiento y sobre las ILPI, se observa que gran parte de la producción científica se concentra en aspectos epidemiológicos, funcionales y organizacionales de estas instituciones, mientras que los aspectos de las dimensiones subjetivas siguen siendo poco explorados, especialmente cuando se trata de la perspectiva de la propia persona mayor, lo que limita la construcción de prácticas asistenciales más sensibles y centradas en la persona^(3,4,5,6,7,8)^. Ante este panorama, se buscó dar voz a las personas mayores que viven en ILPI, con el fin de ampliar el conocimiento sobre las dimensiones subjetivas de este proceso y, así, contribuir a la construcción de prácticas de cuidado más sensibles, humanizadas y alineadas con las necesidades de estas personas.

En este contexto, se formuló la siguiente pregunta de investigación: ¿Cuáles son las experiencias de las personas mayores en el proceso de institucionalización en ILPI? Por lo tanto, el objetivo fue conocer las experiencias de las personas mayores en el proceso de institucionalización.

## MÉTODO

Estudio de enfoque cualitativo, a partir de entrevistas con personas mayores residentes en ILPI, realizado de acuerdo con las directrices de los *Consolidated Criteria for Reporting Qualitative Research* (COREQ).

Los resultados se recopilaron basándose en el Análisis Temático (AT), propuesto por Brawn y Clarke^(11)^.

El proyecto se presentó ante el Comité de Ética e Investigación con Seres Humanos de la institución proponente y obtuvo el dictamen favorable n.º 6.741.923, CAAE 78071324.4.0000.5413. Se informó a las personas mayores sobre el desarrollo del estudio, de conformidad con la Resolución 510/2016 de la Comisión Nacional de Ética e Investigación (CONEP) y, cuando estuvieron de acuerdo, firmaron el Término de Consentimiento Libre e Informado.

La investigación se llevó a cabo en un municipio del interior del estado de São Paulo, Brasil. Se investigaron tres ILPI. El municipio tiene una población de 101 409 habitantes, de los cuales 19 543 son personas mayores, es decir, aproximadamente el 19% de la población general.

Las instituciones se denominaron A, B y C, las cuales se caracterizan por ser asociaciones civiles, de naturaleza jurídica, filantrópicas y sin fines de lucro, que reciben recursos municipales y estatales, así como ayuda de la población local. Están registradas en el Consejo Municipal y Estatal de la Tercera Edad y cuentan con 76, 44 y 47 residentes, respectivamente. Los criterios de admisión en estas instituciones se establecen por resolución judicial, justificada por abandono familiar, solicitud espontánea o por intervención del Consejo Municipal de la Tercera Edad, y algunos de los residentes tienen menos de 60 años, lo cual se debe a la dependencia para las actividades de la vida diaria y a la falta de una red de apoyo social o familiar que garantice su supervivencia en la comunidad. Actualmente, hay una lista de espera para la admisión de nuevas personas mayores en estas instituciones.

Los criterios de inclusión en la investigación fueron: estar institucionalizado desde hace, como mínimo, seis meses, tener 60 años o más, y capacidad comunicativa y cognitiva preservadas, evaluadas a partir del Miniexamen del Estado Mental (MEEM). Se excluyó a las personas mayores con diagnóstico de demencia y a aquellas que, en el momento de la entrevista, se encontraban hospitalizadas o indispuestas por algún problema de salud. El MEEM se aplicó a la totalidad de las personas mayores en una etapa previa del estudio. Entre aquellas que cumplían con los criterios establecidos, se realizó un sorteo para la selección de los participantes. La invitación inicial fue realizada por un profesional del ILPI que tenía una relación previa con la persona mayor; tras obtener su consentimiento, se presentaba al investigador y este procedía a realizar la entrevista. En los casos de rechazo, se procedió a un nuevo sorteo.

Los datos fueron recopilados mediante entrevistas semiestructuradas por el primer autor, enfermero, con maestría, quien ejerce actividades profesionales indirectamente en las instituciones, ya que trabaja en el servicio municipal de vigilancia sanitaria del lugar de la investigación. El entrevistador no tenía relación con los participantes y explicó los motivos de la investigación antes de la recolección de datos. Además, recibió capacitación previa para la realización de las entrevistas. Se siguió un guion con datos de identificación (sexo, edad, tiempo de residencia en la ILPI y preguntas abiertas sobre los motivos para residir en la ILPI, qué cambió en la vida después de ingresar a la ILPI, el significado de estar institucionalizado y cómo es la vida cotidiana en ese lugar).

Las entrevistas se grabaron en audio, se realizaron entre septiembre y diciembre de 2024 en las tres ILPI, sin la presencia de otros participantes o investigadores. Además, tuvieron una duración media de 30 minutos y se dieron por concluidas cuando se observó saturación de los datos, es decir, en el momento en que hubo repetición de los datos y la recolección de nueva información no aportó nada nuevo al objeto de estudio^(12)^. Cabe destacar que no fue necesario realizar entrevistas repetidas, que no hubo entrevista piloto y que el primer autor tomó notas de campo después de cada entrevista. Las entrevistas fueron transcritas íntegramente, inmediatamente después de su realización, por el propio entrevistador responsable de su conducción y no se devolvieron las transcripciones ni los resultados a los participantes. El material empírico se organizó manualmente con la ayuda de *Microsoft Word^®^.* El análisis de los datos se llevó a cabo mediante el análisis temático (AT), que consiste en un método de análisis cualitativo que se propone comprender los patrones derivados de los datos mediante la identificación de temas. El método permite una gran flexibilidad, ya que los temas se extraen de los propios datos, lo que para los autores constituye un proceso creativo y reflexivo, entendiendo la subjetividad del investigador como un recurso necesario, ya que estos no surgen pasivamente de los datos. Cabe destacar, sin embargo, que el enfoque de codificación y desarrollo de los temas debe ser riguroso y sistemático, teniendo en cuenta la necesidad de fluidez y reflexividad del investigador, lo cual debe ocurrir con conocimiento teórico y transparencia^(11)^.

Se añade que la elaboración de los temas se produce mediante sucesivas aproximaciones a los datos y se describe el análisis temático en seis fases, las cuales no deben considerarse de manera lineal, pero es necesaria la exhaustividad en la interacción con los datos, para que se generen percepciones ricas y complejas^(13)^.

La primera fase del análisis temático tiene como objetivo aproximarse a la profundidad y amplitud del contenido. Así, tras la transcripción íntegra de las entrevistas, los seis autores realizaron lecturas repetidas e independientes del material, buscando familiarizarse con el corpus y registrando impresiones preliminares. La segunda fase implica la producción de códigos iniciales a partir de los datos, los cuales representan un contenido semántico o latente que se refiere al segmento o elemento más básico del dato, buscando identificar aspectos interesantes y significativos del texto. En esta etapa, dos investigadores procedieron a la generación inicial de los códigos a partir de la lectura línea por línea de las entrevistas y, a continuación, los códigos fueron revisados, agrupados y refinados, mediante reuniones en línea con los demás autores. En la fase tres, denominada búsqueda de temas, los códigos se organizaron en temas potenciales, los cuales se discutieron colectivamente y se reevaluaron en cuanto a su coherencia interna (homogeneidad) y distinción entre sí (heterogeneidad externa). En la fase cuatro, momento de revisar y refinar los temas, se elaboró un mapa temático, se buscó explicitar la esencia de cada tema, con el fin de asegurar la representatividad de manera consistente del conjunto de datos. A continuación, se definieron y nombraron los temas, en la búsqueda de identificar la esencia de cada uno, y se verificaron los subtemas, asegurando que representaran de manera consistente el conjunto de datos. En la última fase, se llevó a cabo el análisis final y la redacción del informe. En este informe, se seleccionaron extractos de las declaraciones de los participantes y se incorporaron a la narrativa analítica, con el fin de ilustrar y respaldar la interpretación constituida colectivamente por el equipo^(11,13)^. Para mantener el anonimato, las entrevistas se codificaron de acuerdo con la letra asignada a la institución (A, B y C), seguida del orden numérico de realización del 1 al 12.

## RESULTADOS

Se realizaron treinta entrevistas a personas mayores institucionalizadas. De las doce entrevistas realizadas en la institución A, cinco fueron a mujeres y siete a hombres; el tiempo de residencia de la mayoría de los entrevistados era de entre 1 y 5 años y la edad promedio estaba entre los 75 y los 89 años. En la institución B, se realizaron nueve entrevistas, siendo seis participantes hombres y tres mujeres. La mayoría de los entrevistados llevaba entre 1 y 5 años residiendo en el centro y tenía una edad media de entre 60 y 74 años. En el centro C se realizaron nueve entrevistas, cuatro de ellas a hombres y cinco a mujeres. La mayoría de los entrevistados llevaba más de 10 años residiendo en el centro y tenía una edad media de entre 75 y 89 años, según el Cuadro 1.

**Cuadro 1:** Perfil sociodemográfico de las personas mayores institucionalizadas – São Paulo, SP, Brasil, 2025

Institución	Nº Entrev.	Sexo Fem.	Sexo Masc.	Grupos de edad (años)	Tiempo de Residencia (años)
A	12	5	7	75–89	1–5
B	9	3	6	60–74	1–5
C	9	5	4	75–89	>10

Elaboración propia, 2025.

A partir del análisis de los datos obtenidos mediante las entrevistas, fue posible formular cuatro temas: La institucionalización por incapacidad para cuidar y como proceso impuesto por la familia; Las pérdidas derivadas de la institucionalización; El sufrimiento y el malestar en la convivencia institucional; La institución como espacio de cuidado, seguridad y reconstrucción del sentido de pertenencia, tal y como se presentan a continuación.

### Institucionalización por Incapacidad para el Cuidado y como Proceso Impuesto por la Familia

Las personas mayores entrevistadas señalaron que la institucionalización se produjo a partir del momento en que se volvieron dependientes para las actividades de la vida diaria y en que condiciones, como la falta de apoyo, la estructura familiar, las condiciones financieras, además del hecho de vivir solas, les impidieron continuar en sus hogares. En este contexto, algunos fueron institucionalizados conscientes de la necesidad, mientras que otros se sentían contrarios, engañados, con una sensación de falta de voz y, a veces, heridos, ya que la decisión de la institucionalización fue tomada por los familiares. Además, había personas mayores que, aunque no lo deseaban, aceptaron la estancia al reconocer que la familia no podía atender sus necesidades, como se observa en los siguientes comentarios.

*Entonces, ellos no podían preparar el almuerzo ni el café a tiempo, así que me trajeron.* (2B)

*Ah, me quedé solo, perdí a mi esposa. Uno se queda solo y ellos me recogieron y me trajeron aquí. Y yo no... por mí, yo no habría venido.* (A1)

*Sí, mi sobrino fue quien me trajo, yo tampoco lo sabía.* (B4)

*Te sientes mal porque me mintieron, ya sabes. Me mintieron. Dijeron que venían a llevarme al médico. Deberían haber dicho la verdad.* (C2)

*Entonces, vine aquí porque, como puede ver teníamos que pagarle a la cuidadora con mi sueldo. ¿Cómo iba a hacerlo?* (5C)

*Viví 8 años con mis hermanas, pero ya no podían hacerse cargo* [...] *ahora tengo mis límites, ellas se agotaron de hacer todo, por eso vine aquí* [...]. (A9)

### Pérdidas Derivadas de la Institucionalización

La vida de la persona mayor institucionalizada se caracteriza por la pérdida de autonomía e independencia; la ruptura con la identidad y el hogar; una vida cotidiana con rutinas mecanizadas y empobrecidas; y un sentimiento de confinamiento y de no pertenencia. El deseo de estar en su propia casa y tener autonomía para realizar las actividades cotidianas, según su propia voluntad, fue una manifestación presente en el discurso de las personas mayores, lo que genera descontento y tristeza por vivir institucionalizadas. Desde esta perspectiva, señalaron la monotonía de la vida cotidiana en el ILPI, describiendo una rutina impregnada de inactividad y falta de sentido. Se revela, así, que la persona mayor percibe la reducción de su protagonismo y de las posibilidades de elección, así como la ruptura con la identidad y el hogar.

*Creo que uno se queda atrapado. Es que casi no salgo a ningún lado.* (B8)

*Si estuviera en mi casa, comería lo que quisiera. Porque en casa, uno hace lo que quiere. Si pudiera quedarme en mi casa, me quedaría.* (A2)

*No considero este lugar mi hogar, no lo siento como mi hogar* [...] (C8)

*Ah, así es como pasa. Te levantas... te bañan... tomas el café... almuerzas, tomas el café... Y... tomas el café otra vez, almuerzas... Cenás... ¿ves? Y después te vas a dormir.* (A1)

### Sufrimiento e Incomodidad en la Convivencia Institucional

En cuanto a los sentimientos derivados de la institucionalización, los testimonios mostraron un sufrimiento emocional marcado por la soledad y el distanciamiento de los familiares; entornos colectivos con factores estresantes y falta de privacidad; convivencia difícil con otras personas mayores; y baja identificación con el entorno institucional. Alegaron que en el ILPI había personas muy diferentes y enfermas, con trastornos mentales, con las que era difícil convivir; que el ambiente era ruidoso y que el hecho de estar tres en la habitación era incómodo y agobiante, lo que llevaba al distanciamiento, a la percepción de inadecuación y a la falta de pertenencia al lugar.

*Es triste. Estar lejos de la familia. Porque si le digo al señor que es algo bueno, no lo es. Hay veces que estoy triste, lloro, tengo que llorar, ya sabe.* (B6)

*Amigo, te digo la verdad, estoy aquí, pero no estoy contento, ¿sabes?* (A4)

*Es una institución para ancianos enfermos. Entonces... Como eso es difícil, hay gente difícil. Entonces, no le presto atención al ambiente.* (2C)

*Hay algunos que tienen problemas mentales, es difícil. No es que no me caigan bien. No funciona.* (B7)

*Entonces, la mayor parte del tiempo me quedo adentro. No me siento muy a gusto, hay gritos, es un caos. Además, todo es un poco loco, gritos, caos, llantos, insultos.* (A2)

*Es que estamos tres en la habitación. Ya sabes lo que es. Tres en la habitación. Me vuelvo loco, no tengo aire.* (A5)

### La Institución como Espacio de Cuidado, Seguridad y Reconstrucción del Sentido de Pertenencia

Las personas mayores entrevistadas valoraban el apoyo institucional, ya que lograban mejoras en su salud y bienestar gracias a los cuidados ofrecidos; la institución les proporcionaba tranquilidad y protección frente a la falta de apoyo y de condiciones de las familias, lo que se revelaba en experiencias marcadas por el abandono, los conflictos y el sufrimiento; ellos construyeron nuevos vínculos y un sentimiento de pertenencia al entorno; con ello, se adaptaban y sentían satisfacción con la rutina institucional. Se observó, así, que la institución era considerada como un camino de protección y cuidado, ofrecía seguridad y estabilidad, ya que contaba con recursos importantes para mantener la supervivencia, como alimentación a la hora indicada, cuidados de higiene, ropa lavada, medicamentos, distracciones como la radio y la televisión, además de la atención que recibían de los empleados.

*Mejoró bastante, porque aquí tengo una alimentación adecuada, duermo tranquilo, hay que quedarse aquí, que es mejor.* (A12)

*Ah... ¿El trato que nos dan? Ah... excelente, ¿no?* (A1)

*Me gusta que nos atiendan aquí a la hora indicada. Es bueno. Entonces, comida, ropa lavada.* (B3)

*En cuanto a la salud, también mejoró bastante. Muchas cosas buenas. Excelente.* (A9)

*Me quedo aquí escuchando la radio en la silla de ruedas y también veo la televisión. Me gusta todo lo que pasa. Nada me molesta.* (C5)

*No tengo familia. Mi familia son las personas de aquí del albergue.* (B2)

*Porque no tenía paz, vivía en una agonía terrible. No podía dormir por la noche. Y mi hija llegaba borracha. Me quedaba pensando: ¿qué va a pasar hoy?* (C4)

*Aquí es mi casa ahora; cuando vivía con mi hermano y mi cuñada, ellos salían y me dejaban sola en casa con sed y hambre.* (B6)

*Ah, vivía con mi hija y no me llevaba bien con ella, todo me resultaba difícil, por eso pensé: “Me voy a vivir al asilo”, vine y me gustó. Para mí, es mejor que en casa* [...] (A9)

## DISCUSIÓN

En las experiencias de las personas mayores institucionalizadas, se observó que el ingreso a la institución ocurría en diferentes circunstancias, predominantemente asociadas a un proceso de fragilización funcional y a la insuficiencia de apoyo familiar. La dependencia para realizar las actividades de la vida diaria, sumada a la sobrecarga familiar y a la escasez de recursos, constituye un conjunto de factores que con frecuencia culmina en la institucionalización^(14)^. Muchas veces, este proceso no fue participativo, configurándose como una decisión familiar mediada por límites concretos en la capacidad de cuidado. Aunque puede representar una solución práctica para la familia, la institucionalización fue vivida por las personas mayores como una imposición, acompañada de frustración, dolor y sensación de pérdida de autonomía^(15)^.

En este contexto, la autonomía se destacó como una dimensión relevante para comprender las experiencias relatadas. Más allá de la capacidad funcional, se trata de un principio ético que sustenta la dignidad humana. Cuando la decisión de institucionalización se toma sin la participación de la persona mayor, se produce una ruptura con la autodeterminación que puede repercutir en la identidad y en la forma en que el sujeto percibe su lugar en el mundo. En este sentido, el proceso de institucionalización no se limita al cambio de residencia, sino que implica la reconfiguración de los roles sociales, la reorganización de la vida cotidiana y el redimensionamiento de la identidad. Por lo tanto, es necesario que los profesionales que atienden a estas personas tengan claro el significado de la autonomía y cómo facilitarla de manera eficaz^(16)^.

La institucionalización representa un gran impacto en la vida de la persona mayor, ya que proporciona a los residentes límites claramente definidos y reglas que reorganizan la vida cotidiana. Implica, inicialmente, un proceso de desposesión de roles, pérdida de autodeterminación y falta de privacidad y, a continuación, la reconstrucción gradual de nuevos roles, la mayoría de ellos minuciosamente regulados por las normas de la institución. Dicho proceso puede interpretarse como una transición forzada entre modos de existencia, en la que la identidad construida anteriormente se desarticula parcialmente para dar lugar a una identidad institucional. Con la pérdida de la autodeterminación, algunos se sienten frustrados y descontentos^(17)^.

La vida cotidiana en la institución, tal como se expresa en los relatos de los entrevistados, a menudo implica prácticas que violan la dignidad y los derechos de las personas mayores, las cuales terminan naturalizándose y percibiéndose como necesarias para el funcionamiento de la institución.

Esta naturalización contribuye a que la persona mayor sea vista más como un objeto de trabajo que como un ser humano dotado de singularidad, lo que favorece la pérdida de subjetividad^(18)^.

En el contexto de la institucionalización, se destacó un fuerte sentimiento de no pertenencia, que se manifiesta como exclusión, rechazo y aislamiento. Estos sentimientos debilitan la autoestima, comprometiendo la realización personal, el logro del bienestar y, en algunos casos, favoreciendo la aparición de sufrimiento psicológico y enfermedades mentales^(19)^. La falta de pertenencia, desde esta perspectiva, expresa una ruptura con los vínculos sociales que daban sentido a la existencia anterior a la institucionalización.

Los hallazgos de este estudio, que evidenciaron la institucionalización como un proceso frecuentemente impuesto, impregnado de pérdidas, sufrimiento emocional y fragilización de la identidad, dialogaron directamente con los supuestos de la modernidad, tal como lo discute Zygmunt Bauman. En la modernidad líquida, las relaciones sociales se han vuelto más vulnerables, efímeras y pragmáticas, lo que repercute directamente en la forma en que la sociedad aborda la vejez y la dependencia. A medida que las relaciones sociales se vuelven más frágiles y desechables, el cuidado del otro, especialmente de aquel que exige tiempo, paciencia y responsabilidad, tiende a percibirse como una carga, y no como un compromiso ético^(9,10)^.

En este contexto, el concepto de “adiaforización de la conducta humana” contribuye a comprender la banalización del sufrimiento de las personas mayores institucionalizadas. La adiaforización, según Bauman, se refiere al desplazamiento de determinadas acciones del ámbito de la responsabilidad moral, lo que permite que se tomen decisiones como la institucionalización sin la participación de la persona mayor sin que generen culpa o una reflexión ética profunda^(10)^. Así, decisiones como la institucionalización sin la participación de la persona mayor pasan a justificarse como necesarias o inevitables, sin que haya reflexión ética ni profundización sobre sus consecuencias subjetivas. Esto se expresó en los discursos de los participantes que relataron haber sido engañados, privados de elección y alejados abruptamente de la convivencia familiar.

Además, la lógica del consumismo, la productividad y el rendimiento, centrales en la modernidad líquida, entra en choque directo con las necesidades del envejecimiento, que exigen cuidado continuo, escucha, tiempo e inversión emocional. Quien no se ajusta a la lógica de la eficiencia pasa a ser socialmente desplazado hacia espacios de segregación funcional. Así, la persona mayor, al no corresponder ya a los ideales de plena autonomía y eficiencia, pasa a ser percibida como un cuerpo excedente, lo que contribuye a la naturalización de la exclusión del espacio familiar y social, reforzando los sentimientos de no pertenencia identificados en este estudio^(9,10)^.

Así, el proceso de institucionalización plantea a la persona mayor muchos desafíos. El período de adaptación se vuelve casi insoportable, provocando sentimientos de tristeza y abandono, lo que puede llevarla a la depresión^(20)^. En muchas instituciones, la falta de infraestructura y de recursos humanos, sumada a una atención puramente asistencialista, con escaso incentivo para desarrollar el potencial de la persona mayor y su libertad de elección, puede aumentar el grado de dependencia, el aislamiento y la falta de perspectivas para una vida activa y de calidad^(21)^.

Sin embargo, las ILPI desempeñan un papel importante ante las múltiples necesidades que presentan las personas mayores, quienes no siempre cuentan con el apoyo adecuado para el mantenimiento de la salud y la vida, tal como se observó en los comentarios de los entrevistados, quienes consideran que en la institución encontraron mejores condiciones de supervivencia, en comparación con las experiencias anteriores de escasez de recursos financieros o de apoyo familiar. No obstante, esta función protectora no elimina las vulnerabilidades que atraviesan el contexto institucional. Las personas mayores residentes presentan demandas múltiples y complejas, que exigen una atención continua, calificada e interdisciplinaria. En este escenario, especialmente las de carácter filantrópico, enfrentan limitaciones estructurales, financieras y organizativas que repercuten directamente en la calidad de la atención ofrecida^(22)^.

La literatura señala que la falta de cualificación de los profesionales en las ILPI constituye un factor central en este proceso, lo que contribuye a la precariedad de la atención y compromete la calidad de vida de los residentes. Además, la insuficiencia de formación específica y de educación permanente puede favorecer el deterioro de la salud de los propios trabajadores que lidian con demandas complejas y crecientes, a menudo sin el apoyo adecuado^(23,24)^.

En Brasil, aunque existen políticas públicas sólidas, como el Estatuto de la Persona Mayor, aún se observa la insuficiencia de acciones concretas del Estado en la oferta de cuidados a la población de edad avanzada. Como consecuencia, las Instituciones de Estancia Prolongada para Personas Mayores (ILPI) de gestión privada han crecido en el país, operando a menudo sin la debida regulación^(25,26)^.

A diferencia de lo que señalan el presente estudio y otros sobre las deficiencias de las ILPI en Brasil, se cita, como ejemplo, lo observado en Austria, ya que, en general, la calidad de vida en los hogares de personas mayores es elevada, superando, en algunos aspectos, a la de las personas mayores que viven en su domicilio con características sociales y de salud similares, incluso considerando la necesidad de mejorar los cuidados relacionados con la autonomía^(27)^.

En ese país, las personas mayores reciben ayuda económica según el grado de dependencia, priorizando la atención domiciliaria con el apoyo de cuidadores informales. Además, se respeta el deseo de la persona mayor de ser institucionalizada o no^(28)^. El apoyo brindado a las personas mayores y, en consecuencia, las mejores condiciones de vida, también se observan en países como Finlandia, Noruega, Suecia y Dinamarca, dado que se han realizado inversiones elevadas en servicios de atención^(29)^.

Se destaca, así, que la mejora de la calidad de vida de las personas mayores depende de inversiones efectivas que den lugar a la implementación de políticas públicas, comenzando por las existentes, asegurando acciones que promuevan la autonomía, la participación social y la valorización de la subjetividad y la identidad personal. La valoración de la subjetividad reconoce que los seres humanos son vulnerables. Esta categoría incluye a las personas mayores y a sus familiares, así como a los profesionales de la salud que prestan cuidados en ILPI^(30)^.

Por último, se reconoce que, aunque el presente estudio aporta reflexiones sobre las necesidades de las personas mayores institucionalizadas, presenta como limitación el hecho de haberse realizado en tres ILPI de un único municipio, lo que limita la generalización de los hallazgos a otras localidades, ya que la estructura, las prácticas de atención, las políticas locales y las condiciones de los residentes pueden variar. Además, al tratarse de un enfoque de cuestiones delicadas relacionadas con la sensación de abandono, los conflictos y el sufrimiento, es posible que algunas personas mayores hayan optado por no revelar aspectos dolorosos de sus experiencias.

## CONSIDERACIONES FINALES

El presente estudio, al dar voz a las personas mayores institucionalizadas, puso de manifiesto que la institucionalización se produce, predominantemente, en contextos de incapacidad para el cuidado en el hogar y de insuficiencia de apoyo familiar, y que a menudo se vive como un proceso impuesto y marcado por pérdidas, especialmente de autonomía, vínculos y subjetividad. Al mismo tiempo, se reveló como un espacio de protección y acceso a condiciones básicas de supervivencia, configurando una experiencia ambivalente, situada entre el sufrimiento y el cuidado, la pérdida y la seguridad.

Los resultados de este estudio permitieron afirmar que la institucionalización de las personas mayores no puede entenderse únicamente como consecuencia de la dependencia funcional o de la insuficiencia de las políticas públicas, sino también como expresión de un modo de organización social que debilita los vínculos, relativiza las responsabilidades y banaliza el sufrimiento humano. Tal ambivalencia refleja los límites y las posibilidades de las ILPI en el contexto brasileño.

Ante esto, se refuerza la necesidad de fortalecer las políticas públicas y las redes de apoyo que amplíen las alternativas al cuidado institucional, así como la cualificación de las prácticas en las ILPI, con énfasis en la promoción de la autonomía, la escucha activa y el cuidado centrado en la persona. Invertir en la formación de los profesionales y en la construcción de entornos institucionales humanizados constituye un paso esencial para asegurar experiencias más dignas a las personas mayores.

## Data Availability

Todo el conjunto de datos que respalda los resultados de este estudio se publicó en el propio artículo.
